# Detecting Key Structural Features within Highly Recombined Genes

**DOI:** 10.1371/journal.pcbi.0030014

**Published:** 2007-01-26

**Authors:** John E Wertz, Karen F McGregor, Debra E Bessen

**Affiliations:** 1 Department of Microbiology and Immunology, New York Medical College, Valhalla, New York, United States of America; 2 Microbiology Research Group, Thames Valley University, London, United Kingdom; University of Texas, United States of America

## Abstract

Many microorganisms exhibit high levels of intragenic recombination following horizontal gene transfer events. Furthermore, many microbial genes are subject to strong diversifying selection as part of the pathogenic process. A multiple sequence alignment is an essential starting point for many of the tools that provide fundamental insights on gene structure and evolution, such as phylogenetics; however, an accurate alignment is not always possible to attain. In this study, a new analytic approach was developed in order to better quantify the genetic organization of highly diversified genes whose alleles do not align. This BLAST-based method, denoted BLAST Miner, employs an iterative process that places short segments of highly similar sequence into discrete datasets that are designated “modules.” The relative positions of modules along the length of the genes, and their frequency of occurrence, are used to identify sequence duplications, insertions, and rearrangements. Partial alleles of *sof* from Streptococcus pyogenes, encoding a surface protein under host immune selection, were analyzed for module content. High-frequency Modules 6 and 13 were identified and examined in depth. Nucleotide sequences corresponding to both modules contain numerous duplications and inverted repeats, whereby many codons form palindromic pairs. Combined with evidence for a strong codon usage bias, data suggest that Module 6 and 13 sequences are under selection to preserve their nucleic acid secondary structure. The concentration of overlapping tandem and inverted repeats within a small region of DNA is highly suggestive of a mechanistic role for Module 6 and 13 sequences in promoting aberrant recombination. Analysis of *pbp2X* alleles from *Streptococcus pneumoniae,* encoding cell wall enzymes that confer antibiotic resistance, supports the broad applicability of this tool in deciphering the genetic organization of highly recombined genes. BLAST Miner shares with phylogenetics the important predictive quality that leads to the generation of testable hypotheses based on sequence data.

## Author Summary

Microbial genes that accumulate large amounts of nucleotide sequence diversity through lateral exchanges with other microorganisms are often central to understanding key interactions between the microbe and an ever-changing host or environment. Proper sequence alignment of multiple gene alleles is an essential starting point for many of the tools that provide fundamental insights on gene structure and evolution, and allow scientists to develop hypotheses on biological processes. However, for some of the most interesting genes, a good quality alignment can be impossible to attain. We introduce a new software program, BLAST Miner, for analyzing genes that cannot be well-aligned. It relies on identifying small gene segments having high levels of sequence homology, irrespective of their relative positions within the different genes. Genes encoding a drug-resistance determinant and a target of host immunity are used as examples to demonstrate the application of BLAST Miner, and a potentially novel mechanism for generating genetic change is uncovered. This new bioinformatics tool provides an avenue for studying genes that are intractable by most other analytic approaches.

## Introduction

Rapidly evolving genes are among the most biologically intriguing, yet they are also among the most difficult to analyze. The arms race between host and pathogen, fueled by strong selection pressures, can yield very high levels of sequence variation in certain microbial genes. Diversifying selection is often imposed on the microbial pathogen by the adaptive immune response of the host or through antibiotic therapy.

Genetic change can arise through mutation and recombination. These processes often lead to insertions and deletions (indels). Intragenic recombination between divergent sequences can lead to mosaic-like structures. Unless genetic regions of extensive sequence heterogeneity are interspersed with sufficiently long stretches of highly conserved sequence, multiple-sequence alignments can be difficult or impossible to attain. Yet, an accurate and reliable alignment is an essential starting point for many of the analytic tools that provide fundamental insights on gene structure and evolution, such as phylogenetics.

Among the many microorganisms that exhibit extensive genetic diversity are pathogenic streptococci, including S. pneumoniae and S. pyogenes. These organisms cause a wide variety of diseases, ranging from mild to severe, and infect humans throughout the world. A hallmark feature of S. pneumoniae is the widespread emergence of resistance to penicillin within the past 40 years. Resistance is conferred by enzymes known as penicillin-binding proteins (PBPs), which primarily function as transpeptidases active in cell wall biosynthesis, but have evolved a lower binding affinity for the inhibitory drug [1,2]. Until the recent advent of sequence-based typing [3], strains of S. pyogenes were defined by serological-based–typing schemes targeting proteins present on the bacterial cell surface in numerous, antigenically distinct forms [4]. Included among these is serum opacity factor (SOF), which also plays a role in virulence. Both streptococcal species are characterized by relatively high rates of genetic recombination resulting from horizontal gene transfer events [5]. Highly diversified microbial genes often play a key role in the pathogenesis of infectious diseases.

Many alleles of *pbp* genes have been identified, and for the most part their sequences can be readily aligned, allowing for detailed structural analysis. However, attempts to produce a multiple sequence alignment of *sof* alleles have been confounded by their extensive sequence heterogeneity. In an effort to achieve a better understanding of the underlying structural organization of *sof,* a new bioinformatics tool—BLAST Miner—was developed. The well-studied *pbp2x* alleles of S. pneumoniae were also assessed by BLAST Miner, in order to test the broader application of this software to highly diversified genes.

## Results

### Sequence Alignments of *pbp2x* and *sof* Alleles

Multiple-sequence alignments of alleles corresponding to the *pbp2x* locus of S. pneumoniae and the *sof* locus of S. pyogenes are graphically depicted in Figure S1; the *pbp2x* and *sof* loci encode PBP-2x and SOF proteins, respectively. Although *pbp2x* genes display evidence of intragenic recombination and a mosaic-like structure [1,2], the 41 distinct partial alleles of *pbp2x* can be aligned by ClustalW without a single gap. In striking contrast, 45.6% of the positions in the sequence alignment of 139 partial *sof* alleles consists of gaps. The finding of poor sequence alignment of *sof* is not limited to ClustalW; the Muscle and MAFFT programs resulted in an even higher proportion of gaps (Figure S1). The inability to generate a reliable multiple-sequence alignment for *sof* severely limits application of the many tools that are used to assess gene structure, selection, recombination, and phylogeny.

### Analysis of *pbp2x* by BLAST Miner

The BLAST Miner program was developed to better address problems encountered by the lack of accurate sequence alignments for highly diversified genes, such as *sof*. In general terms, this BLAST-based method seeks to identify segments of genes displaying high-sequence homologies, independent of their relative positions along the entire sequence length. To demonstrate the validity and broader application of BLAST Miner, the *pbp2x* gene was first selected for analysis.

The *pbp2x* input data for BLAST Miner consists of 53 partial sequences that are 1,578 bp in length [2], in a FASTA format. The partial alleles encompass the transpeptidase-coding domain, and together the genes encode PBPs that span a wide range of binding affinities for penicillin. BLAST Miner uses the MegaBLAST hit results of an all-ways pairwise comparison (Figure S2), for which the 53 *pbp2x* sequences yielded 10,489 BLAST hit records (Table 1). The parameters for the MegaBLAST step include a word size that is set to 16 bases for the *pbp2x* analysis. Removal of duplicate sequences and their attendant BLAST hit records reduced the total to 6,174, corresponding to the 41 unique *pbp2x* alleles present in the multiple-sequence alignment of Figure S1. In the next step, stringency filtering was performed to remove BLAST hit records that fall below a user-defined percent identity threshold, which was set to 90% for *pbp2x* and yielded 3,220 BLAST hit records (Table 1).

**Table 1:**
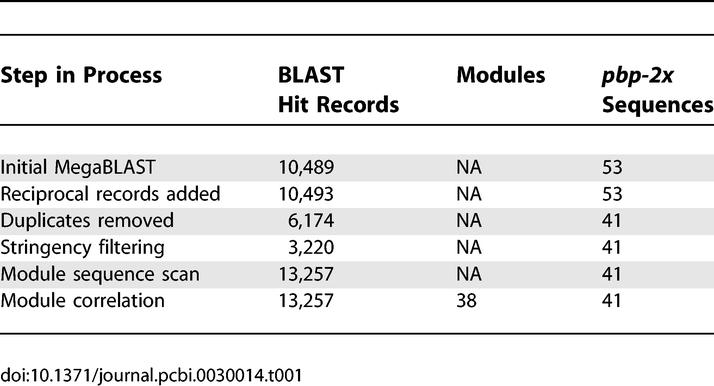
Data Filtering of *pbp2x* Using BLAST Miner

An additional process, denoted as the scanning step, is introduced to reduce the bias that can occur with an extended alignment between two sequences. An extended alignment generates only one BLAST hit record, as opposed to several BLAST hit records having shorter alignment lengths but which together span the same region. The number of bases within the BLAST hit starting region that is used for scanning is user-definable, and was set at 24 for *pbp2x* analysis. The nucleotide (nt) sequences comprising the 3,220 BLAST hit records were used to scan the entire *pbp2x* database for additional exact matches against the starting regions of all original BLAST hits. The scanning step led to an increase in the number of BLAST hit records to 13,252 (Table 1). Since only exact matches (i.e., 100% nt sequence identity) are added, no reduction in stringency results from this process. In effect, this adjustment makes BLAST Miner less sensitive to the size and composition of the sequence dataset.

The fundamental unit that is derived from BLAST Miner is referred to as the module. A module is a dataset comprising >1 segment of nt sequence, corresponding to >1 *pbp2x* allele, whereby each segment is >16 nt in length and has >90% nt identity to at least one other sequence segment assigned to that module. The 16-nt length is based on the word-size setting in MegaBLAST, and the 90% nt identity value reflects the percent identity threshold used for stringency filtering. Segments of sequences that are not included in any BLAST hit record are not assigned to a module and thereby are excluded from further BLAST Miner analyses. Excluded sequences are characterized by their presence in only a single allele and in a nonduplicated form. It is important to emphasize that modules are defined only by their starting position, and that the sequence segments that define a module can vary in length, so long as they exceed the minimum length set by the word size. Module length is indeterminate, but for practical purposes, it is useful to consider the end of a module being the first point at which a subsequent module is defined.

**Figure 1:**
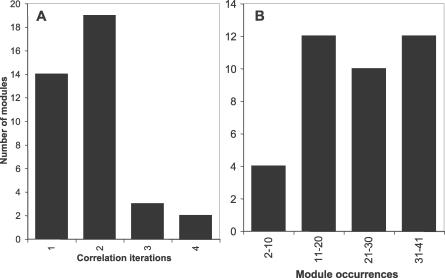
Frequency and Distribution of Modules within Partial *pbp2x* Alleles The *y*-axis indicates the number of modules assigned to each property indicated by the *x*-axis. (A) Number of iterations of the module correlation algorithm used to define each module. (B) Number of module occurrences in the entire dataset of *pbp2x* alleles.

The module correlation algorithm (further described in Materials and Methods, and in Figure S2), was applied to the 13,257 BLAST hit records and yielded 38 modules for *pbp2x* (Table 1). This algorithm assigns discrete segments of nt sequence to a module based on an iterative process that recognizes related BLAST hit records. The extent of nt identity between any two sequence segments that are assigned to the same module can range from 100% to as low as 90% following the first iteration (i.e., equal to percent identity threshold), 81% following the second iteration (i.e., 90% of 90), 72.9% following the third iteration, and so forth. The majority of *pbp2x* modules (33 of 38, or 87%) were assembled by only one or two iterations of the module correlation algorithm (Figure 1A), signifying that all sequence segments assigned to these modules were >81% identical to one another.

The maximum number of occurrences of any one module among the 41 *pbp2x* alleles was 41 (Figure 1B). None of the modules occurred more than once within a given *pbp2x* allele (unpublished data), indicating a lack of intragenic duplications in this dataset.

**Figure 2:**
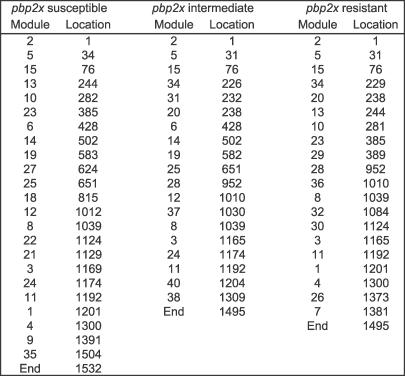
Module Maps of *pbp2x* Alleles The module content (column Module) and nt position of the module start site (column Location) are shown for three *pbp2x* alleles. Accession numbers for the *pbp2x* alleles are X16367, AY0950541, and AY0950557 for the leftmost, middle, and rightmost module maps. The penicillin-resistance phenotypes for the gene products are susceptible, intermediate-resistance, and resistant, respectively [2].

The nt sequence of each *pbp2x* allele can be converted into a module map. Module maps showing the nt start positions for each module are depicted for three *pbp2x* alleles (Figure 2).

### Module Network Structure of *pbp2x*

**Figure 3:**
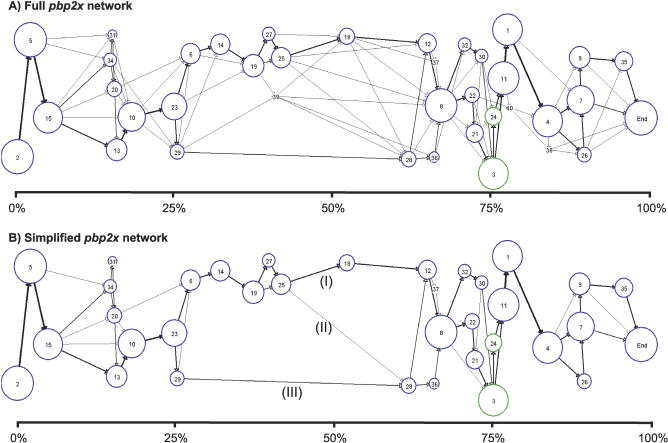
Module Networks of *pbp2x* (A) Shows the complete *pbp2x* module network for 41 *pbp2x* alleles harboring 38 modules. Modules are represented by nodes (depicted as circles), whereby the diameter of the node is proportional to the percent of alleles that harbor it. Node colors represent the number of correlation iterations used to define each node, as follows: blue (more than four iterations), and green (four iterations). Arrows represent the connections between contiguous modules. Arrow thickness is proportional to the frequency with which each connection is observed in the dataset. The module maps of Figure 2 can be traced via arrows through the network graph. (B) Depicts a simplified *pbp2x* network, showing only those modules present in >10% of *pbp2x* alleles (35 modules), and connections that occurred more than two times. Three connection pathways within the central portion of the module network are labeled I, II, and III (see Figure S4 for explanation).

The module maps for all 41 *pbp2x* alleles were combined and graphically represented as one interconnected network of modules (Figure 3). Modules are indicated by the nodes (circles) of the network, whereby the diameter of the node is directly proportional to the number of *pbp2x* alleles harboring that module. Figure 3A shows all 38 modules that were defined for the 41 partial *pbp2x* alleles. The relative position of each node along the *x*-axis reflects the average position of the module start site within the alleles in which the module occurs.

Each *pbp2x* allele represents a walk through the module network graph. Arrows depict the connections between contiguous modules, and the thickness of the arrow is proportional to the number of times that the two connected modules lie adjacent when the complete dataset of 41 *pbp2x* alleles is considered. The direction of the arrowhead depicts the relative order of the two adjacent modules. For the *pbp2x* network, all arrows point in the 5′ to 3′ direction, indicating that there are no rearrangements in the relative order of modules within any of the *pbp2x* alleles. This BLAST Miner finding is consistent with the high-quality multiple-sequence alignment obtained using Clustal W (Figure S1).

The module network structure of *pbp2x* can be used to infer intragenic recombination; however, a systematic method for quantifying recombination remains to be developed. A past history of recombination is strongly suggested by pairs of alleles that follow different pathways into or out of the same node. For example, Module 15 has a single incoming pathway, but five distinct outgoing pathways of connections (Figure 3A, near 5′ end of graph). BLAST Miner is less sensitive at detecting recombination between highly similar sequences as compared with alignment-based methods, since it may fail to detect recombination between pairs of sequences that exceed the percent identity threshold setting. Thus, BLAST Miner provides a conservative estimate of recombination.

Figure 3B depicts a simplified *pbp2x* network, showing only the modules that occur in >10% of the *pbp2x* alleles and connection pathways that occur at least twice. Although even this simplified network is complex, there are several striking features. The central zone of presumed low recombination shows relatively few distinct types of module-to-module connections within the area bounded by Modules 23 and 8. The central region is flanked on both sides by a much higher density of distinct module connections, possibly signifying higher levels of recombination. That the central region encoding the transpeptidase domain may have undergone lower rates of recombination is supported by MaxChi analysis, a standard alignment-based recombination detection method (Figure S3) [6]. In MaxChi, recombination breakpoints are detected near the 3′ end of a conserved sequence, whereas in BLAST Miner the module start site is placed near the 5′ end of a highly homologous region. The actual crossover sites probably lie somewhere in between these two sites, and flank the central portion of the transpeptidase domain.

The central zone of reduced recombination reveals three major pathways of module connections: Module 25 to 18 (pathway I), Module 25 to 28 (pathway II), and Module 29 to 28 (pathway III) (Figure 3B). Of biological relevance is the finding that the three discrete pathways of module connections correlate with drug resistance phenotype, whereby alleles conferring drug susceptibility tend to follow the upper pathway connections (I), alleles conferring resistance tend to follow the lower pathway (III), and alleles that are intermediate in their resistance profile tend to follow pathway II. This finding has further support in a phenogram that is constructed based on the module maps of all 41 *pbp2x* alleles (Figure S4).

The *pbp2x* data indicate that BLAST Miner can uncover key biological relationships between genotype and phenotype. Since an accurate alignment can be readily generated for *pbp2x* alleles, numerous tools are available for gaining an increased understanding of *pbp2x* gene structure, and BLAST Miner does not necessarily provide additional insights on important structural features of the *pbp2x* locus. Instead, the well-studied *pbp2x* genes have served to validate the BLAST Miner application.

### Analysis of *sof* by BLAST Miner

Unlike *pbp2x,* the *sof* alleles of S. pyogenes yield a poor quality alignment that is rich in sequence gaps (Figure S1). BLAST Miner was developed with the goal of providing an analytic tool for otherwise intractable sequence data, such as that found for *sof*. The input data for BLAST Miner analysis of *sof* consists of 249 complete or partial *sof* sequences that had been previously deposited in Genbank or were generated specifically for this study. The input sequences ranged in length from 319 bp for partial *sof* sequences, to 6,386 bp containing the complete *sof* gene and flanking sequence (Table S1).

The all-ways pairwise MegaBLAST analysis of *sof* sequences resulted in ~200,000 BLAST hit records (Table 2). Removal of duplicate records and stringency filtering reduced the number of records to ~78,000, corresponding to 152 unique *sof* sequences. As done for *pbp2x* analysis, the word size was set to 16 and the percent identity threshold was set to 90. The BLAST hit records were used to scan the entire *sof* sequence dataset for additional exact matches, whereby the module alignment length was set to 24 nt. The scanning process increased the number of BLAST hit records to ~228,000. The module correlation process led to the initial assignment of 269 *sof* modules.

**Table 2:**
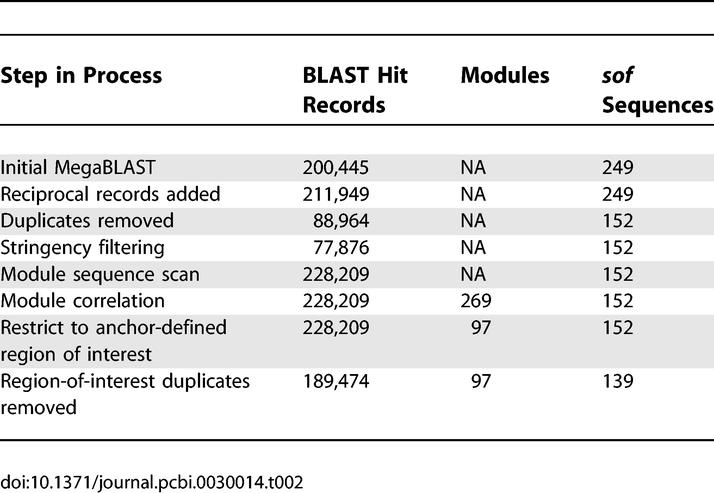
Data Filtering of *sof* Using BLAST Miner

One of the attractive features of BLAST Miner is that the input sequences of the FASTA file do not require prior trimming to a specified length. However, to perform further analysis beyond the initial module assignment, the region of interest within the gene needs to be specified by the user. Anchor modules that define the genetic region of interest are selected via a simple trial-and-error process within BLAST Miner, until the optimal region and/or targeted sequences are bracketed as desired. The anchor modules chosen for *sof* analysis are proximal to the hybridization sites of the oligonucleotide primers used for PCR amplification of the hypervariable region encoding the amino-terminus of the mature SOF protein [7]. The 5′ end anchor module is composed of a single module, designated Module 1; the sequence segments assigned to Module 1 correspond to a portion of the highly conserved signal peptide-coding region. The 3′ end anchor module of *sof*, designated Module End, is a composite of three modules—Modules 10, 51, and 4—because no single module can satisfactorily bracket the region of interest. Modules 10 and 51 are mutually exclusive, whereby either is present in nearly all *sof* alleles. Module 4 was added to the composite anchor Module End to capture the last remaining *sof* sequence. Of the 269 modules that were identified before designation of the anchor modules, ~64% were positioned outside of the anchor-bracketed region. Modules that were not contained within the bracketed region were discarded from the dataset. This reduced the total number of modules for *sof* to 97 (Table 2).

Both the 5′ end anchor Module 1 and the 3′ end anchor Module End were present in 151 of the 152 *sof* sequences remaining in the dataset; one *sof* sequence was truncated and, therefore, was dropped from the dataset. The nt sequences within the anchor-bracketed regions of the remaining 151 *sof* sequences were compared with one another, and 12 duplicate sequences were identified and removed from the dataset (Table 2). The final *sof* dataset contained 139 unique, anchor-bracketed partial *sof* alleles, ranging in length from 329 to 472 bp (Table S1).

### Sequence Composition of *sof* Modules

**Figure 4:**
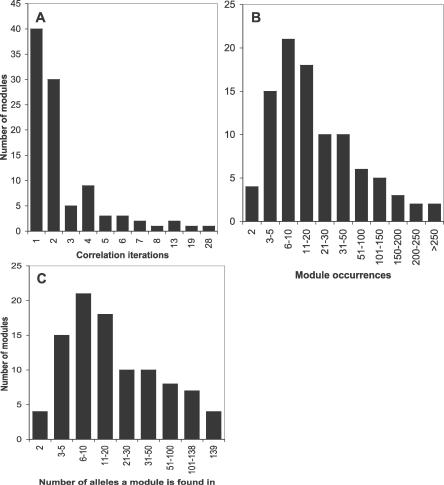
Frequency and Distribution of Modules within Partial *sof* Alleles The *y*-axis indicates the number of modules assigned to each property indicated by the *x*-axis. (A) Number of iterations of the module correlation algorithm used to define each module. (B) Number of module occurrences in dataset. (C) Number of *sof* alleles harboring each module.

Each *sof* module comprises segments of nt sequence, whereby each segment is >16 nt in length and has >90% nt identity of at least one other sequence assigned to that module. The sequence similarity between any two sequence segments assigned to the same module may drop below 90% as the number of iterations of the module correlation algorithm increases. However, all sequences assigned to the same module are related through a network of highly similar intermediates. The distribution of the number of iterations used to define each of the 97 modules of *sof* is depicted in Figure 4A (the complete dataset is presented in Table S2). Most *sof* modules were defined by relatively few iterations, with 78% of modules assembled through <3 iterations, and 42% defined in a single iteration. Modules with the highest number of iterations were Module 6 with 28 iterations, and Module 13 with 19 iterations.

**Figure 5:**
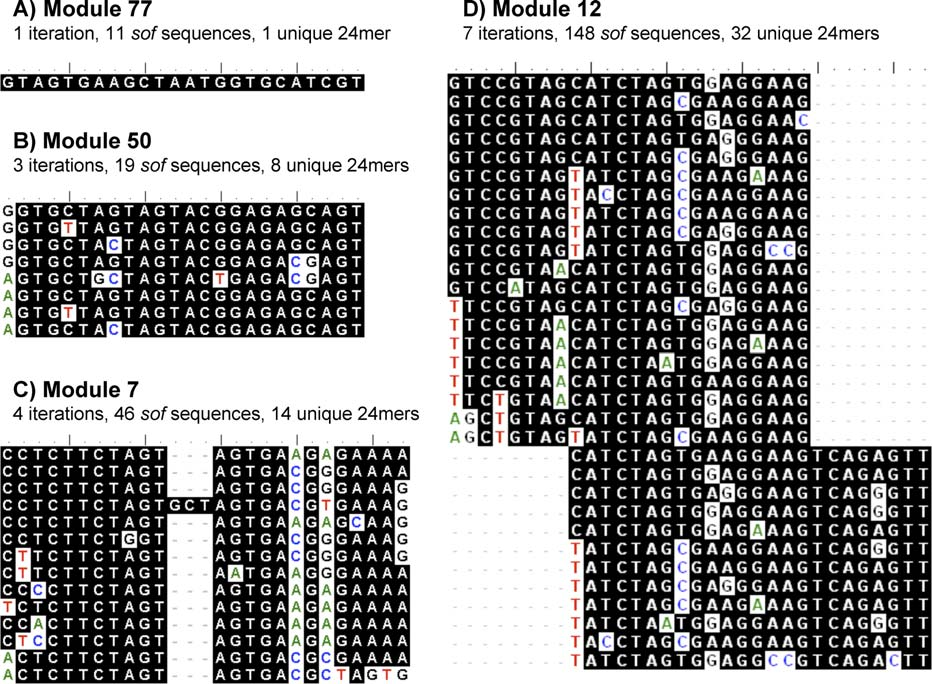
Clustal W Alignments of nt Sequence Segments Segments of *sof* sequences were extracted at the module start site, and included four extra nts upstream from the module start site in order to facilitate alignment. All extracted sequences assigned to a module were aligned using the Clustal W algorithm. The aligned sequences were trimmed to remove the extra 4 nt at the 5′ end, and the 3′ ends were trimmed to yield a total length of 24 nt, allowing for indels. Duplicates of the trimmed sequences were removed from the data shown. Aligned and trimmed sequences are displayed for four modules (A–D). Indicated in each panel are the number of iterations used to define each module and the number of unique trimmed sequence segments. Module 7 provides an example of a 3-bp insertion within the 24-mer backbone. Module 12 (D) provides an example of the module start site slippage that is observed in some high-iteration modules.

Although each *sof* sequence segment assigned to a module has high similarity to more than one other sequence segment within its 5′ end region of 16 nt (i.e., the BLAST hit starting region), the downstream region can vary widely in the extent of sequence similarity. For *sof,* it was observed that high levels of sequence similarity typically extended to >24 nt. The sequence segments of a selected module can be trimmed to 24 bp in length and aligned. Figure 5 provides an example of Clustal W alignments of trimmed sequence segments corresponding to four representative modules (Figure 5A–5D), whereby each module was compiled via a different number of iterations of the module correlation algorithm.

Module 12 sequence segments are defined by seven iterations of the module correlation algorithm, yielding 32 unique 24-mers (Figure 5D). In theory, based on seven iterations, the sequence identity between any two segments assigned to Module 12 can be as low as 47.8%. In actuality, the level of similarity between the 24-mers appears to be much higher. Alignment of the sequence segments corresponding to Module 12 also indicates that the module start site had migrated during the iterative module correlation process by 1 nt to 8 nt, relative to the initial BLAST hit record used in the first iteration. When coupled with the alignment and trimming process for defining 24-mers, the result is two overlapping sets of 24-mer sequence segments offset by 8 nt. Module start site slippage is a common property of high-iteration modules.

### Distribution of Modules among *sof* Alleles

By definition, each of the 97 *sof* modules are represented at least twice within the dataset of 139 partial *sof* alleles. The majority of modules (96%) occur >3 times within the dataset, and 38 modules (40%) occur >20 times (Figure 4B; Table S2). Fifteen modules occur multiple times within the same allele (Table S2); these represent module duplications. The most highly prevalent modules within the *sof* dataset are Modules 6 and 13, with 583 occurrences of Module 6, and 716 occurrences of Module 13. The mean occurrence of Modules 6 and 13 per *sof* allele is 4.2 and 5.2, respectively. Only four of the 97 modules are present within all 139 partial *sof* sequences: the anchor Modules 1 and End (as expected, by definition), and the highly prevalent Modules 6 and 13 (Figure 4C, Table S2). More than 80% of the 97 modules are distributed among at least five *sof* alleles.

The frequency of co-occurrence of modules within individual *sof* alleles was assessed for all possible module pairs. The observed co-occurrence frequency was compared with the null hypothesis (expected), which states that module co-occurrence is random. A chi-squared goodness-of-fit test with the Yates' correction was used to identify module pairs that tend to be either tightly linked or mutually exclusive. Significance values were Bonferroni-corrected for the 4,650 pairwise comparisons performed; the 49 module pairs with adjusted *p*-values < 0.05 are listed in Table S3. Significant, positive values in the deviation from expected co-occurrence were observed for 43 module pairs, indicative of positive linkage. Positive linkage may be the consequence of tight physical linkage or epistatic interactions leading to increased fitness. Negative values in the deviation from expected co-occurrence were observed for six module pairs, indicative of negative linkage or mutual exclusivity.

### Rearrangement in the Relative Order of *sof* Modules

**Figure 6:**
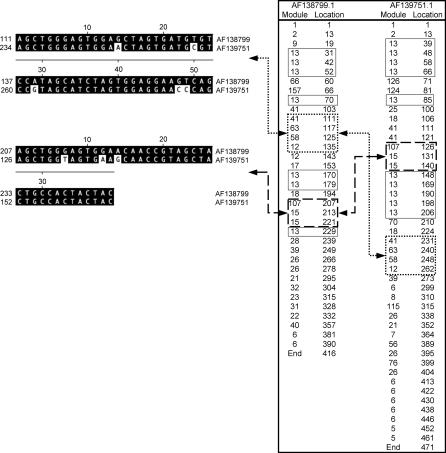
Module Maps and Module Rearrangements Shown (right panel) is the module content (column Module) and nt position of the module start site (column Location) of two *sof* sequences (AF138799 and AF139751); this diagram constitutes a module map. Two major blocks of modules are boxed: short dashes for Modules 41, 63, 58, and 12, and long dashes for Modules 107 and 15. Arrows connecting boxes indicate their relative position within each *sof* allele; the corresponding aligned sequence segment is also shown (left panel). Multiple instances of occurrence of the highly repetitive Module 13 are boxed (thin lines) in order to highlight its position relative to the two major blocks of modules. The module slip threshold parameter (set to 4 nt), which is used in the iterative module correlation process, leads to the identification of sequence segments that are offset by ±4 nt sites; when the offset exceeds twice the module slip threshold parameter, an additional occurrence of that module is declared, even though the start sites of the two modules may be positioned only 8 nt apart. Module start site slippage and discrete blocks composing multiple copies of the same module are depicted in the module maps.

Unlike *pbp2x,* there are numerous examples whereby the relative 5′ to 3′ order of specific modules differ among *sof* alleles. This finding is suggestive of a genetic rearrangement involving co-occurring modules. Since some modules are present multiple times within a single *sof* allele (e.g., Modules 6 and 13), a conservative approach was taken for estimating the number of module pairs having undergone rearrangements in their relative order, whereby only the 5′-endmost position of each module in a given *sof* allele is used to ascertain the relative module order. The relative order of modules in co-occurring pairs was assessed for all 139 partial *sof* alleles. Eighty-seven module pairs displayed a switch in their relative order (unpublished data). Figure 6 (right panel) shows module maps for two *sof* alleles, and illustrates a reversal in the relative order of two blocks of modules. Thus, the data provide evidence for a history of aberrant recombination involving *sof* genes.

### Module Network Structure of *sof*

**Figure 7:**
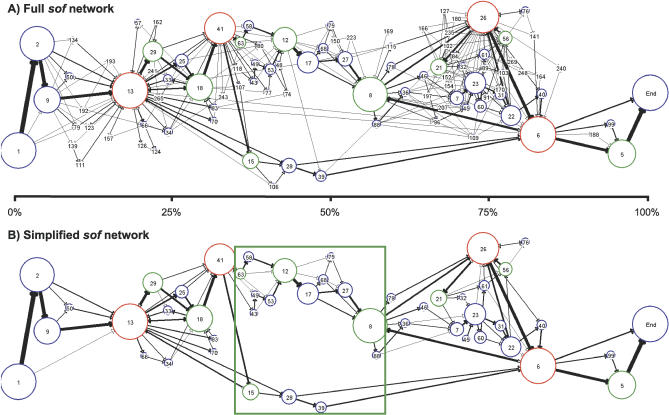
Module Networks of *sof* (A) Shows the complete *sof* module network for 139 *sof* alleles harboring 97 modules. Modules are represented by nodes (depicted as circles), whereby the diameter of the node is proportional to the percent of alleles that harbor it. Node colors represent the number of correlation iterations used to define each node, as follows: blue (fewer than four iterations), green (four to eight iterations), and red (more than eight iterations). Arrows represent the connections between contiguous modules. Arrow thickness is proportional to the frequency with which each connection is observed in the dataset; for this diagram, one pixel is equivalent to ten connections. Tandem repeats of modules are not displayed in the network, but are shown in the module maps of each allele. The module maps of Figure 6 can be traced via arrows through the network graph. (B) Depicts a simplified *sof* network, showing only those modules present in >10% of *sof* alleles (49 modules), and connections that occurred more than two times. The boxed area highlights a region of reduced recombination, and illustrates two discrete pathways of connections within the network.

The module maps of all 139 *sof* alleles are graphically summarized as an interconnected network of modules (Figure 7). Figure 7A depicts all 97 modules that were identified among the 139 partial *sof* alleles, whereas Figure 7B depicts only the 49 modules that occur in >10% of the *sof* alleles. Modules 1, 6, 13, and End are present in all 139 (100%) *sof* alleles (Figure 4C), and, therefore, these four modules correspond to the largest nodes. The relative position of each node along the *x*-axis reflects the average distance of the module start position from the 5′ and 3′ ends of the anchor-bracketed region, whereby distance is based on the first occurrence of the module within each *sof* allele containing that module.

In contrast to *pbp2x* (Figure 3), the *sof* network graph displays backward-oriented arrowheads (Figure 7). This finding is indicative of a genetic rearrangement that changes the relative order of two modules within a single *sof* allele. Alternatively, backward-facing arrows can arise if a module occurs multiple times within an allele. Since each module is depicted by a single node, a module that is positioned between multiple copies of another module, or is involved in a rearrangement, can yield a series of arrows that form a closed loop. This is readily observed in graphic depictions of the module networks for individual *sof* alleles (Figure S5). In the module network graph for the complete *sof* dataset (Figure 7A), nodes with forward- and backward-facing arrows include Modules 6, 8, 13, 18, and 26, each of which are also highly prevalent among *sof* alleles (Table S2). The high number of both forward- and backward-facing arrows impinging on Module 13 suggests that the corresponding sequence segments have undergone frequent recombination. This idea is further supported by the observation that Module 13 often flanks other modules that have undergone rearrangements in their relative order (Figure 6).

Figure 7B shows a less complex network structure for *sof,* whereby rare modules and connections have been removed. The central portion of the network is relatively devoid of backward-facing arrows (box). This observation suggests that the majority of rearrangements and/or duplications, resulting in shifts in relative module order, have occurred closer to the 5′ or 3′ ends of the *sof* region of interest. Furthermore, within the boxed central region are two dominant pathways of module-to-module connections. The module content of the upper versus lower pathway correlates with module pairs that tend to be mutually exclusive. For example, Modules 12 and 28 have a large negative value in their deviation from their expected co-occurrence (Table S3; corrected *p* < 0.000006), and they occupy similar relative positions along the *x*-axis (Figure 7B). As observed in the central zone of restricted recombination within the *pbp2x* network graph (Figures 3B and S3), the upper and lower pathways of module connections in *sof* may represent two major lineages of *sof* having different selectable phenotypes.

Comparison of the individual module network paths taken by single *sof* alleles highlights the allelic differences (Figure S5A versus Figure S5B). For example, in the 3′ half of the graphs, the upper panel shows a path containing multiple closed loops that involve Module 26, whereas the lower panel shows a continuous linear path through Module 26 to the end of the graph. Closed loops and backward-facing arrows are suggestive of an intragenic rearrangement or duplication.

### Unique Biological Features of *sof*

**Figure 8:**
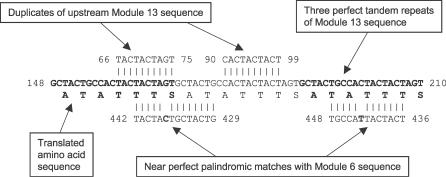
Key Features of Module 6 and 13 Sequences The *sof* sequence AF139751 is presented as an example to show nontandem direct repeats in Module 13 (top strands), tandem direct repeats in Module 13 (middle strand), and complementary inverted repeats in Module 6 (bottom strands). The predicted amino acid sequence of nt positions 148 to 210 demonstrates the Ser-, Thr-, and Ala-codon–rich quality of this region.

Sequence segments corresponding to Modules 6 and 13, as defined by BLAST Miner, exhibit several structural features that may help to explain the biology of *sof*. Along with the anchor Modules 1 and End, Modules 6 and 13 are the only modules that occur in all of 139 *sof* alleles. In addition, Module 6 and 13 sequence segments occur multiple times per *sof* allele (mean of 4.2 and 5.2 times, respectively). However, the 716 occurrences of Module 13 (Table S2) are limited to 289 discrete blocks, many of which contain multiple copies of Module 13; Module 6 displays similar properties. The high occurrence rate for Modules 6 and 13 is probably a consequence of their composition, which is rich in short tandem direct-sequence repeats (Figures 6 and 8).

In addition to tandem duplications, the sequence segments composing Modules 6 and 13 contain inverted repeats (Figure 8). The inverted repeats within Module 13 sequences give rise to complementary pairings both with itself, and in conjunction with Module 6 sequences. The REPuter software tool [8], which computes repeats and palindromes within a single sequence, was used to locate and identify all perfect and near-perfect inverted repeats within the anchor-bracketed region of *sof* sequence AF139751. Table 3 lists all 36 perfect or near-perfect inverted repeats >8 nt in length. Nearly all of the inverted repeats (34 of 36) involve Modules 6 and/or 13 sequences. Of the 18 inverted repeats that involve Module 6 sequences, 16 of the repeats are complementary to Module 13 sequences. The 33 inverted repeats of Module 13 sequence segments form palindromes either with Module 6 segments or with another Module 13 sequence.

**Table 3:**
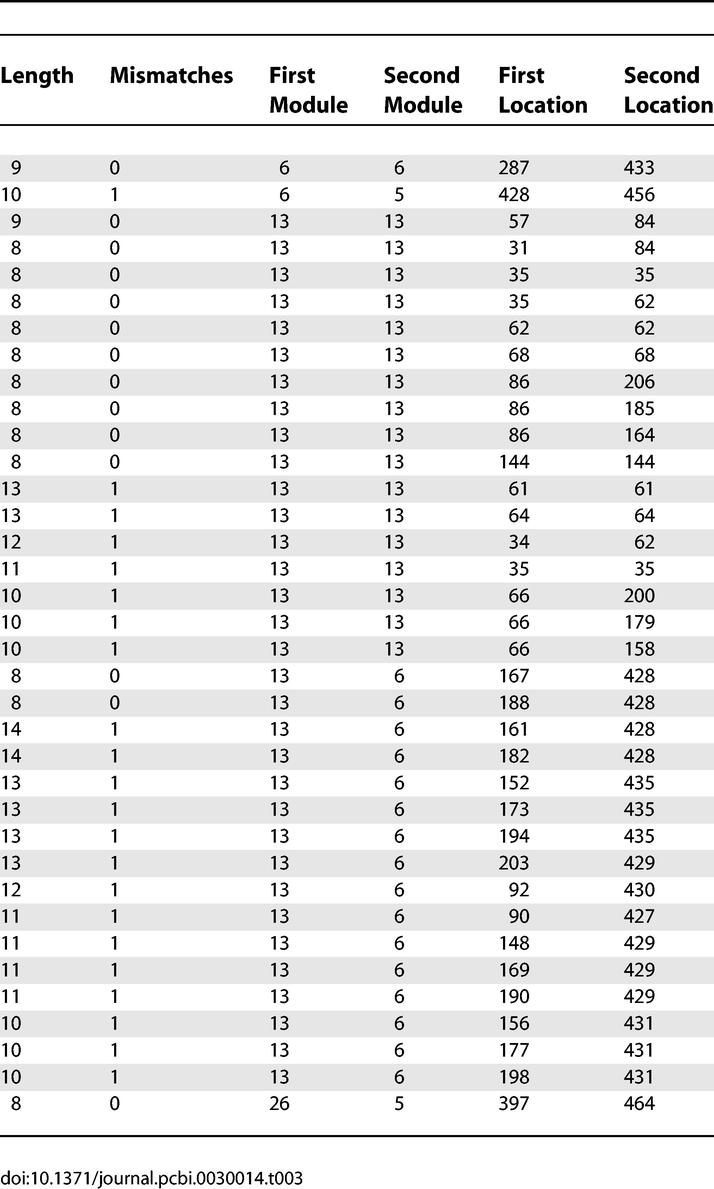
Inverted Repeats in a Representative *sof* Allele (AF139751)

Another notable feature of both Module 6 and 13 sequence segments is that they display strong codon usage bias relative to the complete S. pyogenes genome [9], or when compared with the complete collection of sequences of the 139 partial *sof* alleles (Table S4). For example, the AGU codon for Ser accounts for 79% of Ser-specific codons within the in-frame Module 13 sequences, but accounts for only 23% of the Ser-specific codons present in the complete S. pyogenes genome. Module 13 sequences also have a predicted amino acid sequence that is highly enriched in just a few residues. Seven amino acids account for 96% of the residues within Module 13 sequence segments (Table S4). Top-ranking among these are codons for Thr, Ser, Ala, and Gly, representing 36%, 23%, 17%, and 13% of the codons within Module 13 sequences, respectively. Importantly, the codons themselves form palindromic pairs. For example, AGU encoding Ser is often paired with ACU encoding Thr. Like Module 13, the highly prevalent Module 6 also displays strong codon bias, and is enriched in codons for Thr, Ser, Ala, and Gly that form palindromic pairs.

In summary, BLAST Miner identified Modules 6 and 13 sequence segments within all *sof* alleles and highlighted their unusual structural properties. In-depth examination of the sequences that define Modules 6 and 13 revealed a rich source of short tandem duplications and inverted repeats that form palindromes. The strong codon bias within these palindromic sequence segments is consistent with the idea that strong selection acts at the DNA level to maintain critical biological functions that are dependent on nucleic acid secondary structure. The finding for a high frequency of occurrence of Modules 6 and 13 sequences within partial *sof* alleles, in positions that often flank other modules that have undergone rearrangements or form nontandem direct repeats, suggests that the palindromes may play a mechanistic role in mediating aberrant genetic recombination.

## Discussion

Strong diversifying selection pressures in rapidly evolving microorganisms can yield alleles whose ancestral history is difficult to reconstruct. Although these rapidly evolving genes may represent a small fraction of the genetic content of the microbe, they are disproportionately important when seeking to understand microbial evolution, ecology, and pathogenesis. The inability to obtain an accurate multiple-sequence alignment further heightens the challenge to understand the underlying organization of the gene. The study of such biologically important loci is hampered by the lack of predictive tools that can be used to generate testable hypotheses that address their structure and function.

BLAST Miner differs from many of the predictive tools used for data mining of nt sequence information. Mosaic structures arising from intragenic recombination are often demonstrated via alignment of just a few alleles [7,10,11]. BLAST 2 sequence (bl2seq) is a BLAST-based tool that can be used for detection of duplications and indels [12], but its utility is limited to comparisons of sequences in pairs. There are numerous quantitative methods for detecting probable crossover points [6,13], even when mosaic structures are not obvious. However, these methods use sequence alignments as a starting point. Networked evolution of numerous alleles can be effectively illustrated by splits graphs [14], but split decomposition analysis is also predicated on an accurate multiple-sequence alignment. BLAST Miner does not rely on sequence alignments and instead uses BLAST to search for smaller segments of high-sequence homology among a large set of sequences.

In this report, BLAST Miner was used to analyze genetic regions of ~1.5 kb and ~0.4 kb in length (*pbp2x* and *sof,* respectively). However, it can be applied to a wide size range of sequence length and is limited only by the maximum allowable size of the table generated in the all-ways pairwise MegaBLAST output, which is 2 GB (~20 million records). In theory, BLAST Miner can be used for analysis of pathogenicity islands, phage or other viral genomes, and entire microbial chromosomes. The BLAST Miner software tool can also be used to assess variant loci in lower- and higher-order eukaryotes. Segmentally variable genes, having highly variable regions interspersed among well-conserved stretches [15], may be highly amenable to this analysis.

One of the primary outputs of the BLAST Miner program is the so-called module. Modules are defined strictly in terms of sequence similarity, and biological or evolutionary processes that yield or preserve regions of relatively high sequence identity will tend to enhance the detection of modules at the more stringent parameter settings. Modules start sites are determined by the point at which sequence similarity begins anywhere in the dataset. Another key output of BLAST Miner is a network graph, which simultaneously depicts the relationships between all modules in the entire dataset. The network graph can be used to rapidly identify patterns of potential biological significance that are otherwise difficult to detect via pairwise sequence comparisons. Regions containing conserved or variable sequences can be identified, and regions of the network containing closed loops and backward-facing arrows are good candidates for sites of gene rearrangements or recombination events. Network branch points arise when two or more allele paths enter or leave a module by different routes; this may be a consequence of recombination between alleles, insertions or deletions of module-containing sequence, or simply a high degree of sequence diversity in the region adjacent to a defined module. The latter is suggestive of either purifying selection in the module sequence, or diversifying selection in the adjoining region. However, when interpreting module networks, it is important to bear in mind that module nodes represent only the start sites of regions of sequence similarity; the length of downstream sequence segments of high similarity can vary widely for each allele. Also, highly divergent sequences that are not identified by a BLAST hit are not assigned to a module, and therefore they are not represented in the module graphs.

A central limitation of BLAST Miner lies in the amount of diversity in the test sequences; it is unlikely to be of value for analysis of highly homologous genes. For example, BLAST Miner defined only a single module among 20 partial alleles of *groEL* from *E. coli,* wherein the maximal nt divergence is 3.5% (unpublished data). It is anticipated that BLAST Miner will not be particularly useful for evaluation of these types of housekeeping genes [16,17]. However, its sensitivity and resolution can be appropriately tuned through the user-defined settings for word size, stringency threshold, module alignment length, and the module slip parameter. For example, with the *sof* dataset, increasing the stringency threshold from 90% to 98% nt identity reduces the number of modules from 269 to 204; the maximum number of iterations used to define a module is also reduced (unpublished data). For less diverse genes, the number of modules can be increased by lowering the stringency threshold below 90%, reducing the module slip parameter below 4 nt, or increasing the module alignment length above 24 nt.

The *sof* gene encodes SOF, a recognized virulence factor of S. pyogenes [7,18–23]. SOF is a sortase-anchored surface protein of ~1,000 amino acid residues. SOF contains a fibronectin-binding domain located proximal to the cell wall, a large central domain that interacts with apolipoproteins of mammalian serum and leads to its opacification, and a hypervariable amino-terminal region that appears to be a target of the SOF-serotyping scheme. The serological typing scheme is based on neutralization of the serum opacity reaction by specific antibody. The amino-terminal region of SOF was chosen as the focus of this report, as part of an initial step toward developing a sequence-based typing scheme for *sof* that parallels the widely used approach for *emm*-typing [24]. Although a biological function for the amino-terminal region has yet to be ascribed, this region appears to be under strong diversifying selection, perhaps mediated by a strong host immune response that provides protection against infection.

Identification of Modules 6 and 13 through BLAST Miner may provide a starting point for understanding the genetic mechanisms underlying *sof* diversification. The sequence segments that constitute Modules 6 and 13 are striking in their richness for direct and inverted repeats, and imperfect (quasi) repeats. Both direct and inverted repeats are often sites of genetic rearrangements mediated by DNA mispairing [25]. These misalignments, or slippage errors, are independent of homologous recombination factors such as RecA, and are referred to as aberrant recombination events. Rearrangements mediated by direct repeats can result in genetic duplications or deletions, whereas inverted repeats can lead to inversions of sequence order. These types of genetic changes are evident among the *sof* alleles. Aberrant recombination has been described for other streptococcal species [26,27]. However, the specific mechanisms underlying aberrant recombination in S. pneumoniae and S. suis appear to be distinct from the likely processes governing rearrangements in *sof*, as inferred based on our new knowledge of *sof* gene structure. The strong codon usage bias observed within the Module 6 and 13 sequence segments, and the palindromic codons, are indicative of strong selection pressures that act to preserve the machinery that ultimately gives rise to the genetic changes upon which other selection pressures (e.g., host immunity) can act.

If the Module 6 and 13 sequence segments are hotspots for genetic recombination, they may also be hotspots for small indels to the degree that recombination is error-prone and subject to DNA strand slippage [25]. In this regard, it may be particularly relevant that a relatively high proportion of possible +1 frame shift mutations within Module 6 and 13 sequence segments are predicted to generate a stop codon (i.e., UAA, UAG, or UGA). Specifically, one-third of the codons within Module 13 sequence segments have AA, AG, or GA occupying the first two positions, whereas 66% have uracil in the third position (unpublished data). In the event of a single base (+1) frame shift, an average of 15.2% of the codons within the anchor-bracketed regions of *sof* are converted to stop codons. Overall, Module 13 sequences account for 22.9% of the anchor-bracketed regions of *sof,* yet they contain 32.9% of the stop codons that would occur in the event of a +1 frame shift. This event, in turn, could lead to phase variation in the SOF phenotype. To our knowledge, there is no documentation of phase variation in SOF expression; however, the BLAST Miner findings provide a rational basis for formulating hypotheses to test this biological property. The experimental approach can take the form of screening bacterial variants for a *sof*-positive genotype and a SOF-negative phenotype. In general terms, high-frequency phase variation of a microbial surface protein can be a key part of a survival strategy to escape the host immune response and/or to release the organism from its epithelial attachment site (e.g., promote transmission).

A multiple-sequence alignment is an essential starting point for many of the tools that provide fundamental insights on gene structure, selection, recombination, and phylogeny. However, if recombination is sufficiently high, even tools specifically designed to estimate recombination can exceed their limits. BLAST Miner is a bioinformatics tool that can help provide additional new insights on genes that are intractable with the many tools that rely on an accurate multiple-sequence alignment. It also has the important predictive quality that leads to the generation of testable hypotheses based on sequence data. Analysis of *sof* by BLAST Miner provides evidence for a novel molecular mechanism for generating genetic diversity in *S. pyogenes,* a pathogen characterized by a very high number of genetically distinct clones [28,29].

## Materials and Methods

### Nucleotide sequence data.

The partial nt sequence was determined for *sof* genes following PCR amplification of purified S. pyogenes chromosomal DNA with Primers F2 (5′-GTATAAACTTAGAAAGTTATCTGTAGG-3′) and R3 (5′-GGCCATAACATCGGCACCTTCGTCAATT-3′), according to [7]. Newly identified *sof* alleles were deposited in Genbank and assigned accession numbers DQ450100 to DQ450145.

### BLAST Miner program.

BLAST Miner (version 1.0) is a Microsoft Windows 2000/XP–based stand-alone relational database program written in Delphi (Borland Software, http://www.borland.com). It requires that the Borland Database Engine be installed. The BLAST Miner program, complete with a runtime distribution of the Borland Database Engine, installation instructions, and a user guide, is available for download at http://pantheon.yale.edu/~jw343/blastminer.html. A description of the BLAST Miner algorithm and its applications is provided below and outlined in Figure S2. Figure S6 provides a screenshot of the main window.

### Generating BLAST hit records.

BLAST Miner is designed to use the BLAST hit results of an all-ways pairwise comparison of ~20–200+ nt sequences (e.g., alleles) as input data. All sequences to be compared via BLAST are compiled in a FASTA file; a convenient feature of the BLAST Miner approach is that the nt sequences in the FASTA file need not be trimmed to equivalent lengths or positions at this stage of analysis. The FASTA file is used to generate a BLAST database, using the formatdb program, which is available for download as part of the stand-alone BLAST suite of applications (http://www.ncbi.nlm.nih.gov/BLAST/download.shtml). The stand-alone version of MegaBLAST (version 2.2.12) is used to query the BLAST database with the same FASTA file that was used to generate it, yielding an all-ways pairwise comparison of sequences [30]. The BLAST Miner program handles the interface, and the formatdb and MegaBLAST programs remain hidden. Parameters for the MegaBLAST program include specifying the database, specifying the input and output file names, disabling the complexity filtering, setting the word size (to 16 bases in this study), setting the dropoff value to 10, and specifying a single tab-delimited line per BLAST hit as the output file format.

The MegaBLAST output file is automatically converted into a database table, whereby each BLAST hit is represented by a record that contains the following fields: the name of the query sequence (QueryID), the name of the subject sequence (SubjectID), the percent identity (Identity), the length of the BLAST hit (AlignmentLength), the number of mismatches (Mismatches), the number of gap openings (GapOpenings), the starting and ending position of the BLAST hit in the query sequence (QStart and QEnd), and the starting and ending position of the BLAST hit in the subject sequence (SStart and SEnd).

As a first step in processing the MegaBLAST output data, a reciprocal of each BLAST hit record is generated by reversing the subject and query names, and the subject and query hit locations (QHLs). The reciprocal records are added to the database of BLAST hits. The reciprocal step corrects an artifact in MegaBLAST, and also ensures that all BLAST hits are defined according to both the query and subject sequence names. A query or subject sequence that has 100% nt identity over its entire length to another sequence is considered to be a duplicate, and all records referring to the duplicate sequence are removed from the dataset. User-defined stringency parameters are also applied, in order to remove records that fall below the percent identity threshold. For the analyses of this report, the minimum percent nt identity threshold was set to 90%.

### Database scan with starting regions of BLAST hits.

An additional processing step is included as part of BLAST Miner to render it less sensitive to the size and composition of the sequences in the input dataset. The sequences constituting the BLAST hit records are used to scan the entire database of sequences for additional exact matches against the starting region of the original BLAST hit. The number of bases within the BLAST hit starting region that is used for scanning is user-definable (in the “module length” option of the main window). The scanning step reduces the bias that can occur with an extended alignment between two sequences, which generates only one BLAST hit record, as opposed to several BLAST hit records having shorter alignment lengths but which together span the same region. The scanning step may lead to an increase in the number of BLAST hit records. Since only exact matches (i.e., 100% sequence identity) are added, no reduction in stringency results from this process.

### Module correlation algorithm.

The fundamental unit that is derived from BLAST Miner is referred to as the module. A module is a dataset consisting of segments of nt sequence that have a high percent identity to at least one other sequence segment assigned to that module.

The database table of BLAST hit records is processed by the module correlation algorithm, an iterative process that searches for matches between appropriate parts of the BLAST hit record name assignment, whereby records that share common BLAST hit locations are grouped together. Each BLAST hit record is assigned a QHL and subject hit location (SHL). To assign the QHL value, the BLAST hit records are first sorted by query sequence name, and next sorted by the nt starting position of the BLAST hit within the query sequence. The sorted BLAST hit records are sequentially numbered to yield the QHL assignment, whereby the number is incremented when either (a) the starting nt position exceeds the user-defined module slip threshold parameter or (b) a new query sequence is encountered. The module slip threshold is simply defined as the number of nt bases between starting positions of the sequential BLAST hit locations within a single sequence. In this report, the module slip threshold parameter is set to 4 nt.

Upon completion of assigning the QHL values, the database is sorted by subject sequence name, and subsequently sorted by the starting position of the BLAST hit within the subject sequence. SHL assignments for each BLAST hit record are made according to the numbering process that is described for QHL assignments.

An iterative process is used to assign related BLAST hit records to a module. The iterative process is based on associations between the QHL and SHL numerical values, which reflect the high level of percent nt identity between the query and subject sequence, for the sequence segment that lies immediately downstream of the starting position of the BLAST hit. Since an initial starting point for the iterative process must be chosen, for consistency the most prevalent SHL assignment is selected. All BLAST hit records sharing that SHL value are extracted from the database of BLAST hit records and assigned to the same module (e.g., Module 1). In the next step, all remaining records within the database of BLAST hits that have a QHL value matching any QHL number present among the BLAST hit records belonging to Module 1 are also assigned to Module 1. The process of adding new BLAST hit records to Module 1 is repeated, alternating between QHL and SHL assignments, until no new matching records are found. Thus, at each iteration, sequences are added to a module only if they match an existing BLAST hit record of that module, which in turn is dictated by the percent nt identity threshold parameter.

The entire iterative process is repeated, starting with the most prevalent SHL value remaining among the BLAST hit records that were not extracted by their assignment to the first module; this next dataset of sequence segments constitutes the second module. This process continues until all BLAST hit records are assigned to a module.

### User-defined anchor modules.

Although sequences in the initial FASTA input file need not be aligned or trimmed to a specified length, further sequence analysis requires that homologous regions be compared. Relatively conserved sequence segments that bracket the portion of the gene to be studied are designated as the anchor modules. Due to possible sequence heterogeneity within the bracketing segments, it may be necessary for an anchor module to be defined as a composite of more than one module. Once the anchor-bracketed region is defined, the original input sequences that display 100% identity over the bracketed region are considered duplicates and removed from the dataset.

### Module distribution frequency and order.

The number of occurrences of each module contained within the dataset of alleles or partial alleles, defined as unique anchor-bracketed sequences, can be calculated. If the starting position of a module spans more than twice the module slip threshold parameter, an additional tandem copy of the module is declared. The relative order of each module within each allele can also be ascertained. Differences in the relative order of modules (i.e., rearrangements) can be identified by comparing the module content of all alleles. If a module occurs more than once within an allele, the module positioned closest to the 5′ end of the anchor-bracketed region is used for the purpose of detecting rearrangements. The frequency of co-occurrence, or linkage, of different module pairs can be calculated, and the observed co-occurrence frequency can be compared with the null hypothesis that module co-occurrence is random.

### Module network graphing.

The relationships between all modules of the complete anchor-bracketed dataset can be graphically displayed as a network of modules, defined by nodes that are connected by arrows. The diameter of each node is directly proportional to the percent of sequences in the dataset that contain the module. Arrows represent the connections between contiguous modules. Arrow thickness is proportional to the frequency with which each connection is observed in the dataset. The relative position of each node along the *x*-axis is the average position of the first occurrence of the module within the anchor-bracketed region. The *y*-axis position is arbitrary; it can be adjusted by the user to reposition nodes, in order to minimize their overlap and to enhance visual clarity. Arrow color and node color are also user-definable features. Different node colors can be assigned based on the number of iterations that define a given module. Module network diagrams can be saved in bitmap or enhanced metafile formats.

### Module-based phenograms.

A distance matrix can be constructed from pairwise comparisons of module content of all alleles. The distance score between alleles is calculated based on the presence or absence of shared modules, without regard for differences in the order or number of modules. Scores for modules are weighted, based on their relative frequency in the dataset, such that alleles that differ by a single rare module have smaller distance scores than alleles that differ by a high-frequency module. Weighting was implemented due to the observation that many rare modules arose as a consequence of a very small number of nt substitutions between nearly identical alleles. A neighbor-joining algorithm in the NEIGHBOR program [31] was employed to generate phenograms based on the distance matrices.

### Nt sequence alignment.

The BioEdit sequence alignment editor version 7.0.2 [32] was used for manipulating sequence files and generating sequence identity matrices. The ClustalW algorithm was used for multiple-sequence alignments. Multiple-sequence alignments of the *sof* dataset were also made with the MUSCLE program version 3.6 [33] and the MAFFT program version 5.861 [34].

### Codon usage statistics.

Aggregate codon usage statistics were calculated by concatenating sequences (in frame) and tabulating the collective codon composition, using the EditSeq version 5.52 (DNASTAR, http://www.dnastar.com) program.

## Supporting Information

Figure S1 Multiple Sequence Alignments of *pbp2x* and *sof* Sequences Alignments were made using (A,D) the Clustal W algorithm, (B) MUSCLE, and (C) MAFFT. Each base is depicted by a single pixel: A, green; T, red; G, black; C, blue; gap, grey.
(A–C) Contain multiple alignments of 139 unique partial *sof* alleles, showing a large number of gaps and alignments of low quality.
(D) Contains a multiple alignment of 41 unique partial *pbp2x* alleles, showing no gaps. The partial *sof* alleles were trimmed to only include the sequence between the defined anchor modules (see Results), and ranged in length from 329 to 472 bp. The *pbp2x* alleles display ~19.9% maximal nt sequence divergence. The *pbp2x* alleles of S. pneumoniae used for analysis include all of those reported in [2].
(89 KB PDF)

Figure S2 Steps Taken for Defining Modules The module-defining process, via the module-correlation algorithm, starts with a single BLAST hit in a query sequence. All of the subject sequences that match the initial query hit are assigned to the module. All new hits that were added based on their subject sequence are matched with additional BLAST hits, based on their query sequence. This cross-correlation process continues until no new query sequences can be added. Thus, a module comprises stretches of nucleotide sequence having >90% identity to at least one other sequence within the group; the 90% identity value is user-defined for analysis of *pbp-*2x and *sof*. The iterative process is repeated to generate additional modules, until no unassigned BLAST hit records remain. Each allele can be represented as a series of named modules. The relative position and frequency of modules can also be used to identify duplications, insertions, and rearrangements. Additional details are presented in Materials and Methods.
(25 KB PDF)

Figure S3 Recombination Detected in *pbp2x* via Alignment-Based Methods A simplified module network graph of *pbp2x* (similar to Figure 3B; top panel), is compared with a plot of all statistically significant recombination events as determined by the MaxChi method (bottom panel). Data for the graph in the bottom panel was generated via alignment of all 41 *pbp2x* alleles and subsequent analysis using the MaxChi method in the RDP program (V2 Beta 08; [6]). The number of recombination events with both a beginning and ending point *p*-value < 0.05 are shown (*y*-axis). The *x*-axis depicts the nt site within the alignment. This method, and related methods (e.g., GENECONV), scan aligned sequences and determine recombination breakpoints based on shared polymorphic sites among sequence pairs. Thus, in MaxChi, recombination breakpoints are detected near the 3′ end of a conserved sequence, whereas in BLAST Miner the module start site is placed near the 5′ end of a highly homologous region. The actual crossover sites probably lie somewhere in between the two sites. Importantly, the MaxChi plot displays a central region that is relatively free of predicted crossover points, closely matching the zone of reduced recombination in the module network graph.
(188 KB PDF)

Figure S4 Phenogram Derived from the Module Maps of All 41 *pbp2x* Sequences The corresponding sequences of the three major connection pathways (I, II, and III) indicated in Figure 3B are shown to the right of the phenogram, along with the drug resistance phenotype associated with each *pbp2x* allele. Exceptions to the drug resistance phenotype grouping are marked with red letters designating the observed phenotype.
(53 KB PDF)

Figure S5 Paths of Single *sof* Alleles through the Module Network The module network paths of the same two *sof* sequences (AF139751 and AF138799) shown in Figure 6A and Figure 6B, respectively.
(100 KB PDF)

Figure S6 Main Window of BLAST Miner A screenshot of the main window of BLAST Miner is shown.
(42 KB PDF)

Table S1 Accession Numbers of *sof* Alleles Used for BLAST Miner Analysis (29 KB XLS)

Table S2 Frequency and Distribution of Modules Corresponding to *sof* Alleles (27 KB XLS)

Table S3 Linkage Analysis of Module Pairs (28 KB XLS)

Table S4 Codon Usage Statistics for S. pyogenes and Regions of the *sof* Gene (28 KB XLS)

### Accession Numbers

The GenBank accession numbers for *sof* sequences discussed in this paper are listed in Table S1. The GenBank (http://www.ncbi.nlm.nih.gov/Genbank) accession numbers for *pbp2x* sequences discussed are AY950507 and AY950558, and X16367. Newly identified *sof* alleles were deposited in Genbank and assigned accession numbers DQ450100 to DQ450145.

## Abbreviations

indels: insertions and deletions
PBP: penicillin-binding protein
QHL: querey hit location
SHL: subject hit location
SOF: serum opacity factor
