# Supplementary material for: Detecting Key Structural Features within Highly Recombined Genes
Source: PLoS Comput Biol. 2007 Jan 26;3(1):e14. doi: 10.1371/journal.pcbi.0030014 (PMC1782043; doi:10.1371/journal.pcbi.0030014)

Figure S1

**A) Multiple alignment of 139 unique *sof* partial alleles aligned using ClustalW**

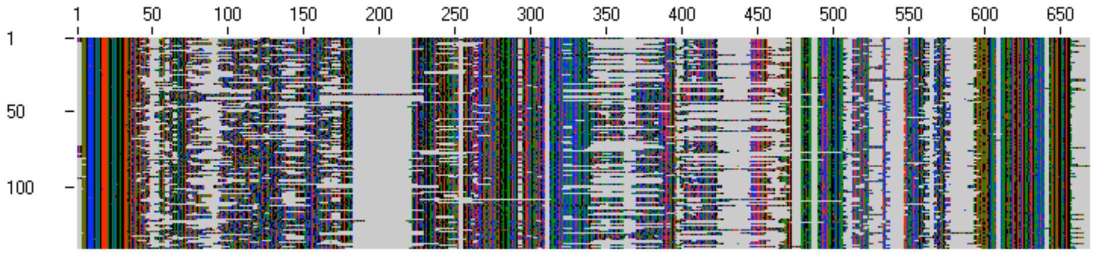


**B) Multiple alignment of 139 unique *sof* partial alleles aligned using MAFTT**

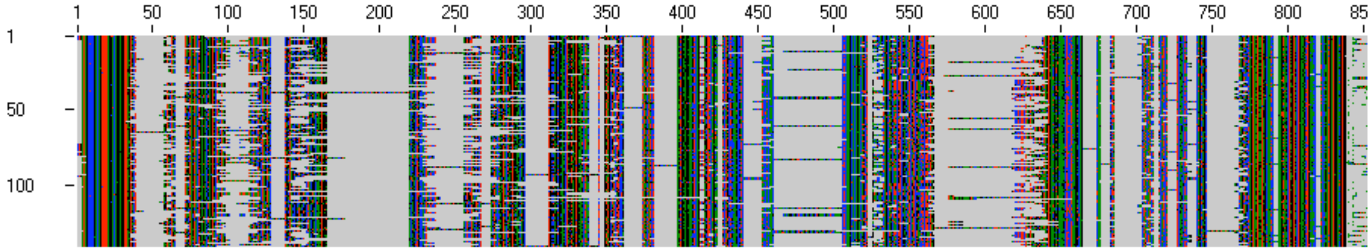


**C) Multiple alignment of 139 unique *sof* partial alleles aligned using MUSCLE**

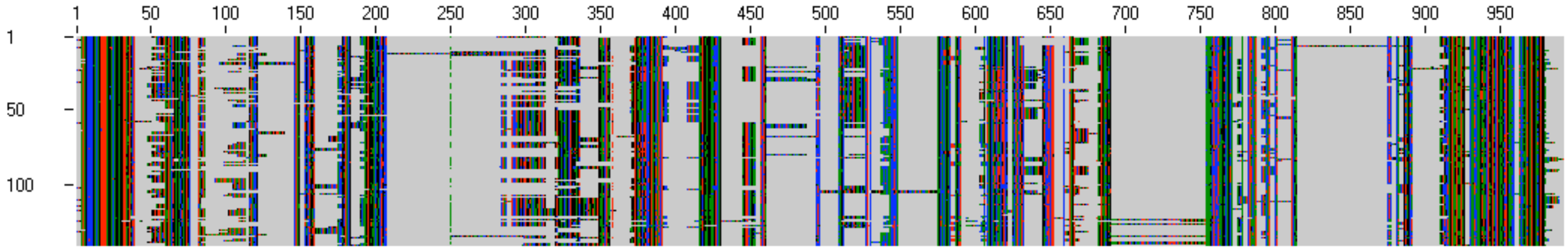


**D) Multiple alignment of 41 unique *pbp2x* partial alleles aligned using ClustalW**

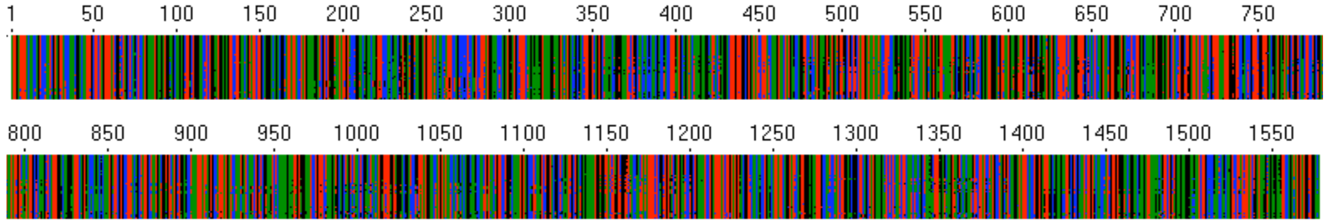

Supplement: Figure S1 — Alignments were made using (A,D) the Clustal W algorithm, (B) MUSCLE, and (C) MAFFT. Each base is depicted by a single pixel: A, green; T, red; G, black; C, blue; gap, grey. (A–C) Contain multiple alignments of 139 unique partial sof alleles, showing a large number of gaps and alignments of low quality. (D) Contains a multiple alignment of 41 unique partial pbp2x alleles, showing no gaps. The partial sof alleles were trimmed to only include the sequence between the defined anchor modules (see Results), and ranged in length from 329 to 472 bp. The pbp2x alleles display ~19.9% maximal nt sequence divergence. The pbp2x alleles of S. pneumoniae used for analysis include all of those reported in [2]. (89 KB PDF) [file pcbi.0030014.sd001.pdf]
