# Supplementary figures and images for: Detecting Key Structural Features within Highly Recombined Genes
Source: PLoS Comput Biol. 2007 Jan 26;3(1):e14. doi: 10.1371/journal.pcbi.0030014 (PMC1782043; doi:10.1371/journal.pcbi.0030014)

Figure S2

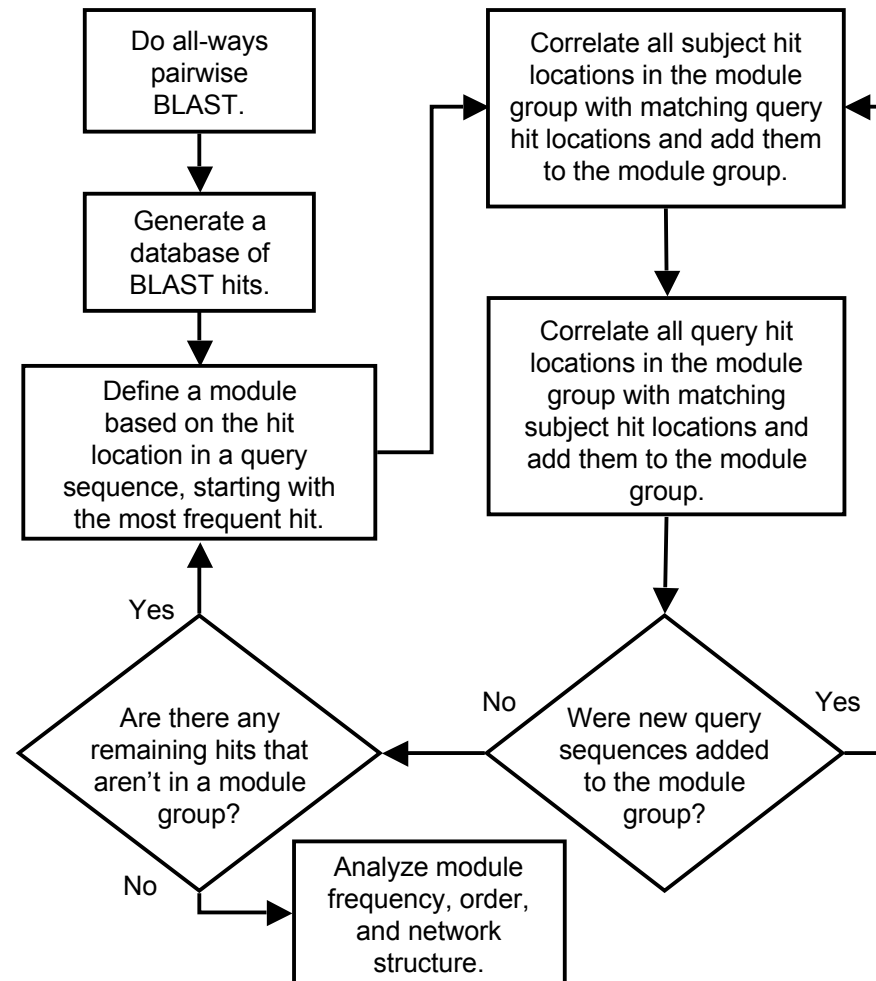

Supplement: Figure S2 — The module-defining process, via the module-correlation algorithm, starts with a single BLAST hit in a query sequence. All of the subject sequences that match the initial query hit are assigned to the module. All new hits that were added based on their subject sequence are matched with additional BLAST hits, based on their query sequence. This cross-correlation process continues until no new query sequences can be added. Thus, a module comprises stretches of nucleotide sequence having >90% identity to at least one other sequence within the group; the 90% identity value is user-defined for analysis of pbp-2x and sof. The iterative process is repeated to generate additional modules, until no unassigned BLAST hit records remain. Each allele can be represented as a series of named modules. The relative position and frequency of modules can also be used to identify duplications, insertions, and rearrangements. Additional details are presented in Materials and Methods. (25 KB PDF) [file pcbi.0030014.sd002.pdf]

Figure S3

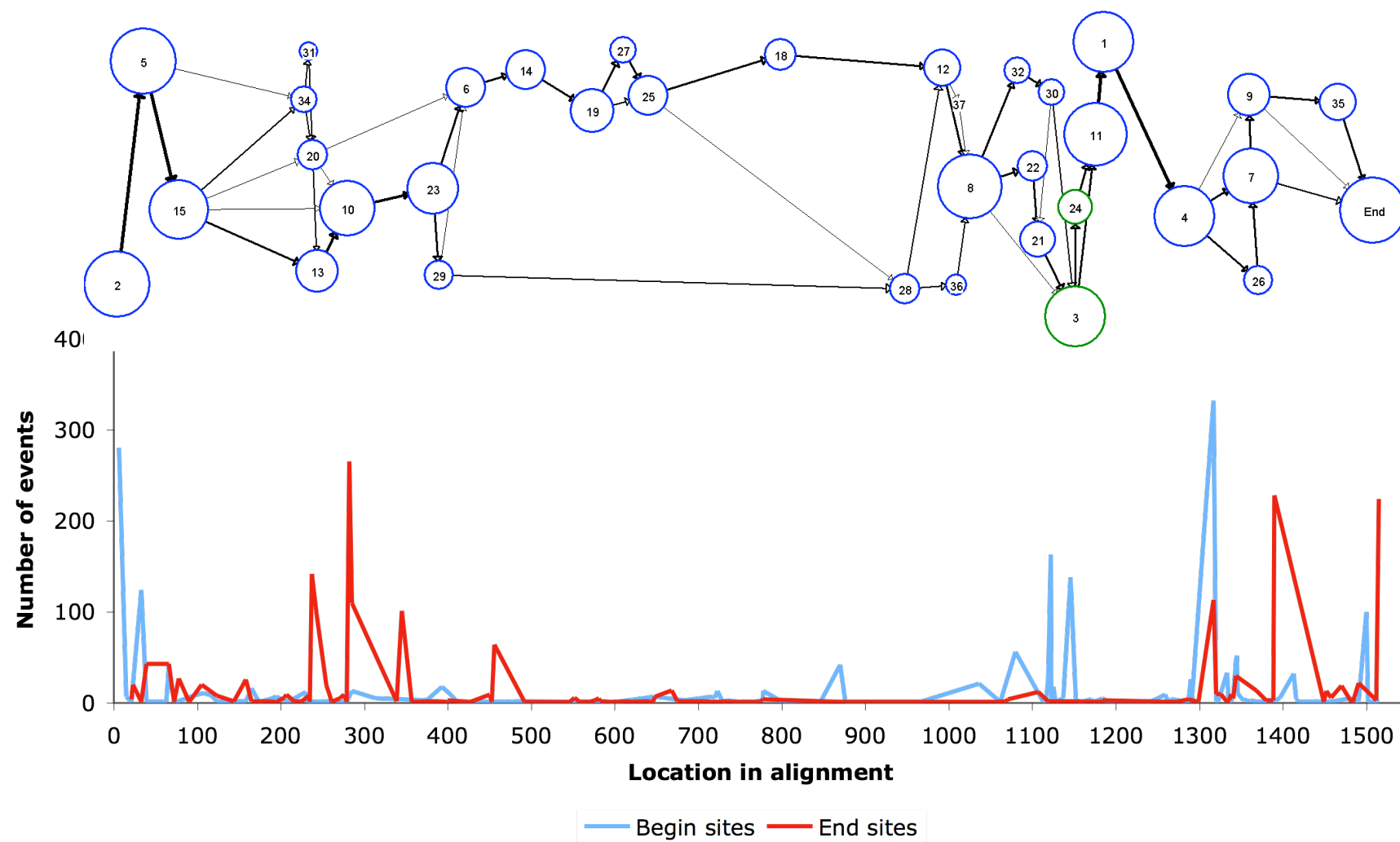

Supplement: Figure S3 — A simplified module network graph of pbp2x (similar to Figure 3B; top panel), is compared with a plot of all statistically significant recombination events as determined by the MaxChi method (bottom panel). Data for the graph in the bottom panel was generated via alignment of all 41 pbp2x alleles and subsequent analysis using the MaxChi method in the RDP program (V2 Beta 08; [6]). The number of recombination events with both a beginning and ending point p-value < 0.05 are shown (y-axis). The x-axis depicts the nt site within the alignment. This method, and related methods (e.g., GENECONV), scan aligned sequences and determine recombination breakpoints based on shared polymorphic sites among sequence pairs. Thus, in MaxChi, recombination breakpoints are detected near the 3′ end of a conserved sequence, whereas in BLAST Miner the module start site is placed near the 5′ end of a highly homologous region. The actual crossover sites probably lie somewhere in between the two sites. Importantly, the MaxChi plot displays a central region that is relatively free of predicted crossover points, closely matching the zone of reduced recombination in the module network graph. (188 KB PDF) [file pcbi.0030014.sd003.pdf]

Figure S4

**A module based phenogram of *pbp2x* mapped to antibiotic resistance phenotype**

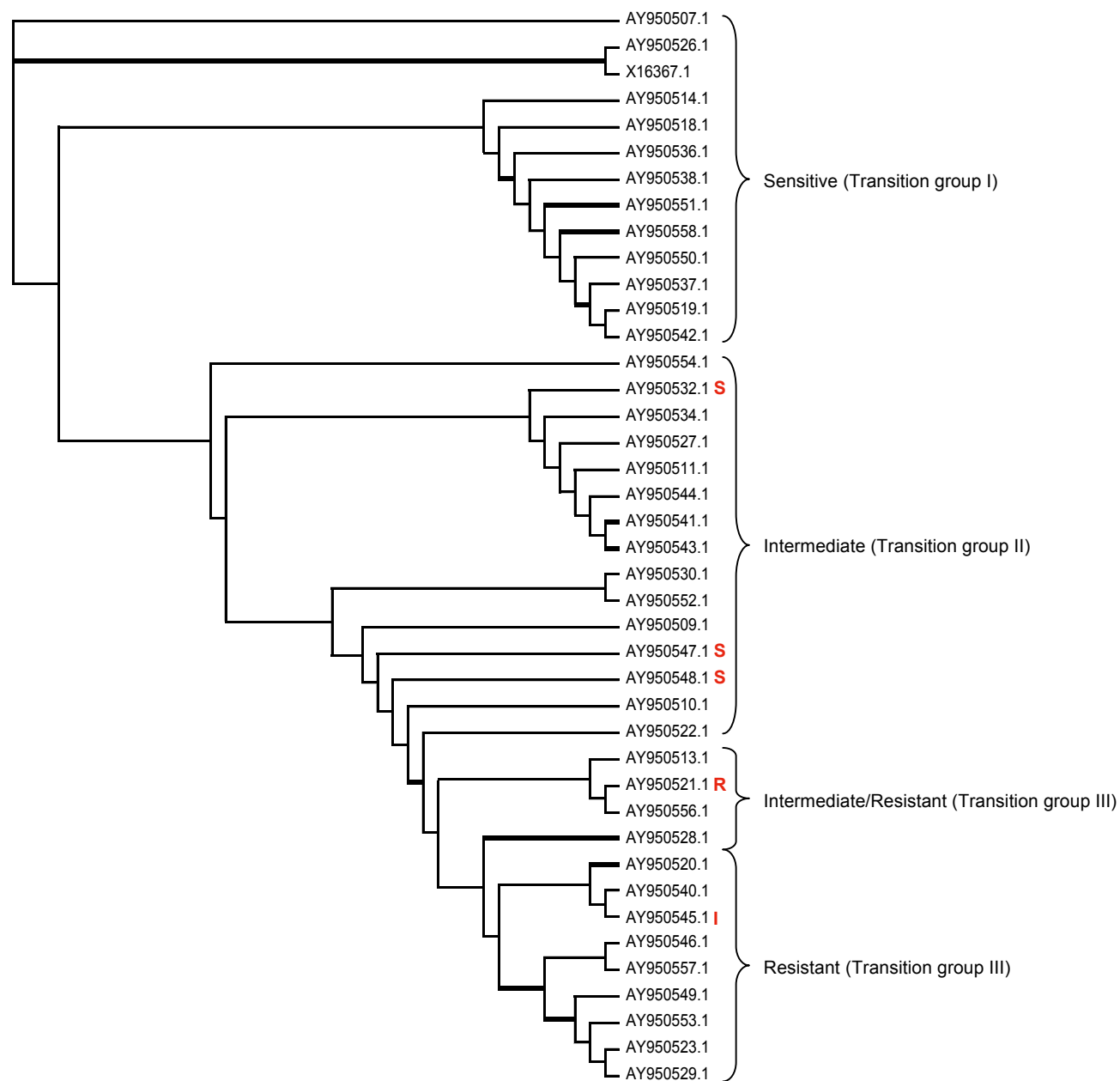

Supplement: Figure S4 — The corresponding sequences of the three major connection pathways (I, II, and III) indicated in Figure 3B are shown to the right of the phenogram, along with the drug resistance phenotype associated with each pbp2x allele. Exceptions to the drug resistance phenotype grouping are marked with red letters designating the observed phenotype. (53 KB PDF) [file pcbi.0030014.sd004.pdf]

Figure S5

A) Network path of *sof* allele AF139751.1

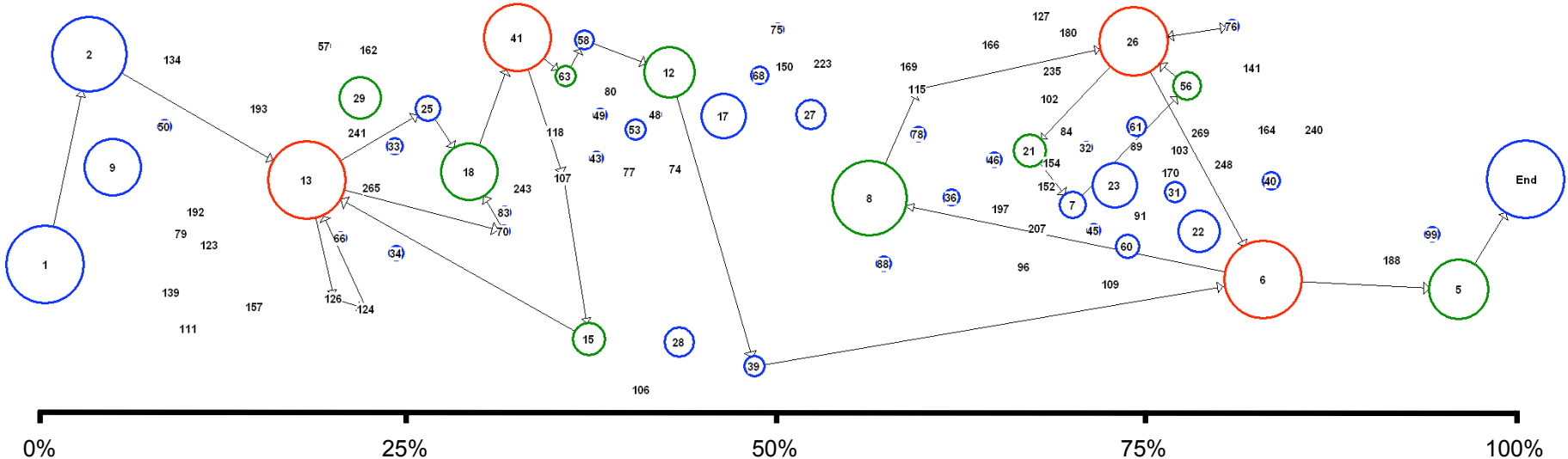

B) Network path of *sof* allele AF138799.1

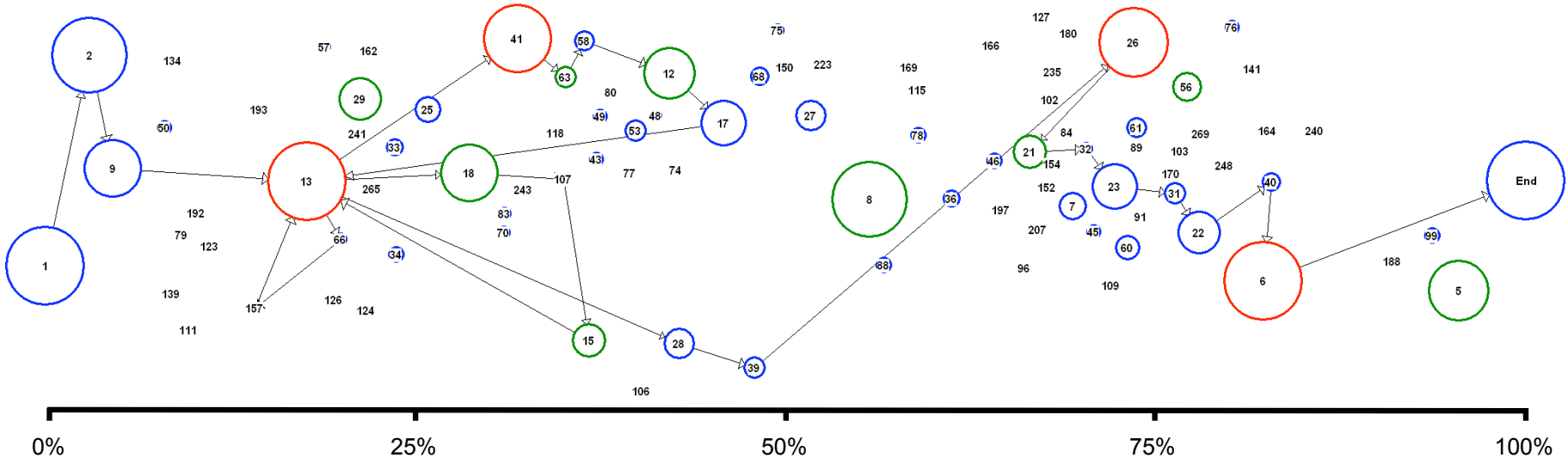

Supplement: Figure S5 — The module network paths of the same two sof sequences (AF139751 and AF138799) shown in Figure 6A and Figure 6B, respectively. (100 KB PDF) [file pcbi.0030014.sd005.pdf]
