# Supplementary material for: Detecting Key Structural Features within Highly Recombined Genes
Source: PLoS Comput Biol. 2007 Jan 26;3(1):e14. doi: 10.1371/journal.pcbi.0030014 (PMC1782043; doi:10.1371/journal.pcbi.0030014)
Supplement: Figure S6 — A screenshot of the main window of BLAST Miner is shown. (42 KB PDF) [file pcbi.0030014.sd006.pdf]

Figure S6

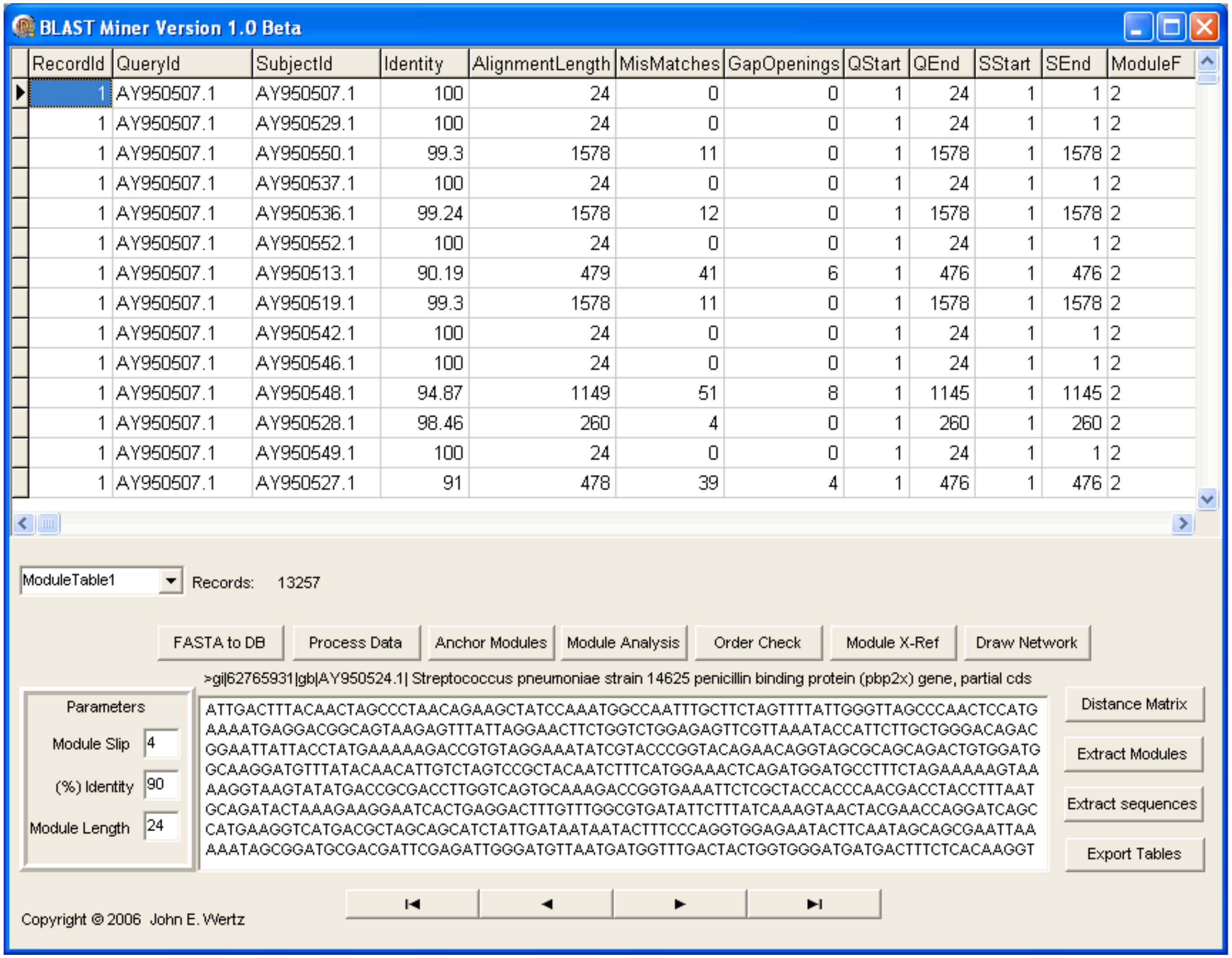
BLAST Miner Version 1.0 Beta
RecordId QueryId SubjectId Identity AlignmentLength MisMatches GapOpenings QStart QEnd SStart SEnd ModuleF
1 AY950507.1 AY950507.1 100 24 0 0 1 24 1 1 2
1 AY950507.1 AY950529.1 100 24 0 0 1 24 1 1 2
1 AY950507.1 AY950550.1 99.3 1578 11 0 1 1578 1 1578 2
1 AY950507.1 AY950537.1 100 24 0 0 1 24 1 1 2
1 AY950507.1 AY950536.1 99.24 1578 12 0 1 1578 1 1578 2
1 AY950507.1 AY950552.1 100 24 0 0 1 24 1 1 2
1 AY950507.1 AY950513.1 90.19 479 41 6 1 476 1 476 2
1 AY950507.1 AY950519.1 99.3 1578 11 0 1 1578 1 1578 2
1 AY950507.1 AY950542.1 100 24 0 0 1 24 1 1 2
1 AY950507.1 AY950546.1 100 24 0 0 1 24 1 1 2
1 AY950507.1 AY950548.1 94.87 1149 51 8 1 1145 1 1145 2
1 AY950507.1 AY950528.1 98.46 260 4 0 1 260 1 260 2
1 AY950507.1 AY950549.1 100 24 0 0 1 24 1 1 2
1 AY950507.1 AY950527.1 91 478 39 4 1 476 1 476 2
ModuleTable1
Records: 13257
FASTA to DB
Process Data
Anchor Modules
Module Analysis
Order Check
Module X-Ref
Draw Network
>gi|62765931|gb|AY950524.1| Streptococcus pneumoniae strain 14625 penicillin binding protein (pbp2x) gene, partial cds
Parameters
Module Slip 4
(%) Identity 90
Module Length 24
ATTGACTTTACAACTAGCCCTAACAGAAGCTATCCAAATGGCCAATTTGCTTCTAGTTTTATTGGGTTAGCCCAACTCCATG
AAAATGAGGACGGCAGTAAGAGTTTATTAGGAACTTCTGGTCTGGAGAGTTCGTTAAATACCATTCTTGCTGGGACAGAC
GGAATTATTACCTATGAAAAAGACCGTGTAGGAAATATCGTACCCGGTACAGAACAGGTAGCGCAGCAGACTGTGGATG
GCAAGGATGTTTATACAACATTGTCTAGTCCGCTACAATCTTTCATGGAAACTCAGATGGATGCCTTTCTAGAAAAAGTAA
AAGGTAAGTATATGACCGCGACCTTGGTCAGTGCAAAGACCGGTGAAATTCTCGCTACCACCCAACGACCTACCTTTAAT
GCAGATACTAAAGAAGGAATCACTGAGGACTTTGTTTGGCGTGATATTCTTTATCAAAGTAACTACGAACCAGGATCAGC
CATGAAGGTCATGACGCTAGCAGCATCTATTGATAATAATACTTTCCCAGGTGGAGAATACTTCAATAGCAGCGAATTAA
AAATAGCGGATGCGACGATTCGAGATTGGGATGTTAATGATGGTTTGACTACTGGTGGGATGATGACTTTCTCACAAGGT
Distance Matrix
Extract Modules
Extract sequences
Export Tables
Copyright © 2006 John E. Wertz
